# Multi-phases of islet beta-cell function change in type 2 diabetes mellitus and its influencing factors

**DOI:** 10.3389/fendo.2025.1602796

**Published:** 2025-10-10

**Authors:** Jin Cheng, Jun Li, Yaping Xin, Dongming Zhang

**Affiliations:** Department of Metabolism and Endocrinology, The Second Affiliated Hospital of Zhengzhou University, Zhengzhou, Henan, China

**Keywords:** beta-cell, C-peptide, HOMA-β, insulin, type 2 diabetes

## Abstract

**Aims:**

Based on cross-sectional and follow-up data, we aimed to explore the continuous long-term pattern of beta-cell function change in type 2 diabetes and to analyze the relevant influencing factors.

**Materials and methods:**

Data from 2898 type 2 diabetic subjects were retrospectively analyzed. Islet beta-cell function was evaluated by the homeostasis model assessed index (HOMA-β). The pattern of association between HOMA-β and disease duration coverup of 50 years were explored using non-linear regression approaches. Findings were replicated in longitudinal follow-up data from multi-centers. Influencing factors of both residual HOMA-β level and HOMA-β decline rate were investigated.

**Results:**

We identified a model including three clear phases of HOMA-β change: an initial ascending phase over 4.2 years from diagnosis (3.34% change per year [95%CI 0.04, 6.52]), followed by a phase of exponential fall up to 20.9 years from diagnosis (-3.04% change per year [95%CI -3.78, -2.29]) and thereafter a low and plateau phase (0.17% change per year [95% CI -0.72, 1.05]). Longitudinal follow-up data verified this model. Higher BMI (OR = 1.103 [95%CI 1.047, 1.161]), UA (OR = 1.003 [95%CI 1.001, 1.005]), metabolic Syndrome (OR = 1.526 [95%CI 1.021, 2.279]) and lower HbA1c (OR = 0.695 [95%CI 0.627, 0.771]) levels were independently associated with higher residual HOMA-β level. Earlier diagnosis (Coefficient=0.0009 [95%CI 0.0002, 0.0016]) was independently associated with faster HOMA-β decline.

**Conclusions:**

Beta-cell function change in the course of type 2 diabetes was nonlinear with multi-phases. Targeting the factors that affect different phases would contribute to the protection of the disease progression.

## 1 Introduction

Type 2 diabetes mellitus is characterized by insulin resistance and a progressive loss of islet beta-cell function (1). Although both factors contributed to the pathogenesis, decreased beta-cell function and beta-cell mass are the predominant factors of disease progression, and are the typical hallmark of the overt-diabetes (1). In fact, studies including the United Kingdom Prospective Diabetes Study (UKPDS) (2) have revealed that despite its latent nature, beta-cell function impairment was indeed an early event in the course of the disease. As such, only 50% of beta-cell function remained at diagnosis and continued declining at a rate of 5% annually (2). Moreover, the impaired beta-cell function is closely related to oral treatment failure, blood glucose fluctuation, microvascular or macrovascular complication, and an increased mortality (3–6). Therefore, protecting beta-cell function is an essential goal in the prevention and treatment of type 2 diabetes (7).

A comprehensive understanding of the natural history of type 2 diabetes, especially the trajectory of beta-cell function change, would provide a significant theoretical basis for preventing and treating the disease. Some studies have explored this issue and reported that rates of beta-cell decline varied (2, 8–11). However, most of these studies focused on Western populations and were virtually small-sample, short follow-up observations with highly selected subjects. Furthermore, none of these studies revealed the continuous long-term pattern of beta-cell function change. Besides, the prevalence of type 2 diabetes in China has increased over 10-fold in the past 40 years, which now has the largest type 2 diabetes population in the world (12). Therefore, data from the Chinese type 2 diabetes populations would provide significant support.

To address these gaps, this study recruited 2898 Chinese type 2 diabetes patients and detected their beta-cell function change trajectory over 50 years. The relevant influencing factors were also analyzed. Thus, this study would help further clarify the model of beta-cell function change, identifying optimal intervention timing and targets.

## 2 Materials and methods

### 2.1 Subjects

#### 2.1.1 Cross-sectional cohort

**Figure 1:**
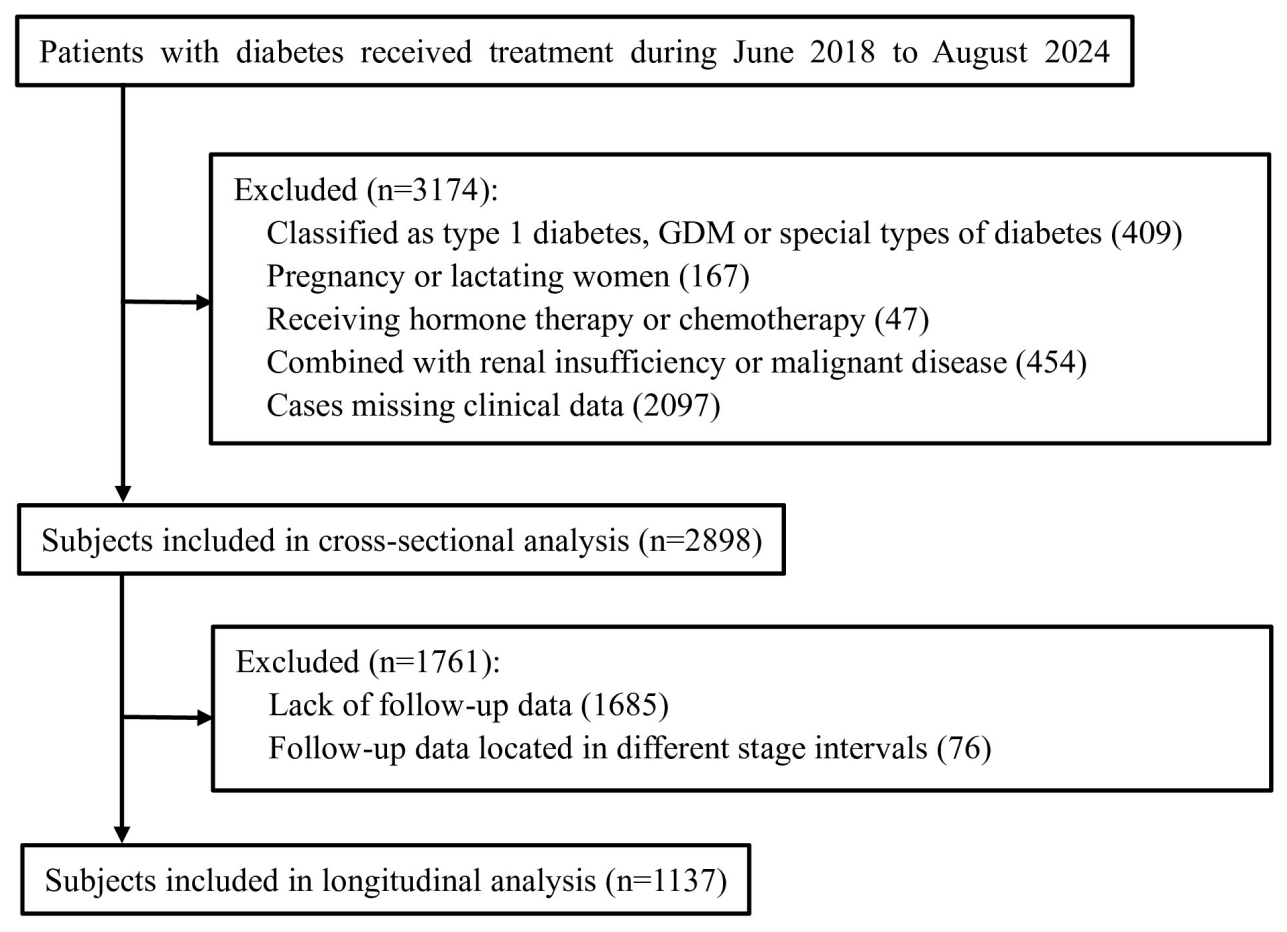
Flow-chart for enrollment.

This study was conducted in accordance with the guidelines of the Declaration of Helsinki (as revised in 2008). All patients provided informed consent to participate in the study. The study protocol was approved by the Research Ethics Committee of the Second Affiliated Hospital of Zhengzhou University (No. KY2024134). We retrospectively analyzed data from 6072 subjects with diabetes who received treatment in the Department of Endocrinology, Second Affiliated Hospital of Zhengzhou University, from June 2018 to August 2024. Participants were enrolled if they fulfilled the following criteria: (a) diabetes diagnosed according to the 1999 World Health Organization (WHO) criteria (13); (b) clinically classified as type 2 diabetes; (c) testing negative for diabetes-associated autoantibodies, namely glutamic acid decarboxylase antibody (GADA), islet cell antibody (ICA), and insulin autoantibody (IAA) (for patients with a history of insulin use, IAA was not included in the analysis). Exclusion criteria included: (a) classification as type 1 diabetes mellitus [defined as an acute-onset, insulin-dependent disease at diagnosis caused by beta-cell destruction, according to guidelines from WHO (13) and the American Diabetes Association (ADA) (14)], gestational diabetes, or other special types of diabetes; (b) pregnancy or lactation; (c) receipt of hormone therapy or chemotherapy; (d) comorbidity with renal insufficiency [since blood C-peptide levels in patients with renal function impairment are artificially elevated (15)] or malignant disease. A total of 4995 patients met the above criteria. Subjects lacking measurements of fasting C-peptide (FCP), fasting plasma glucose (FPG), disease duration, or other relevant data were excluded. Finally, 2,898 subjects were included in the cross-sectional analysis (**Figure 1**).

#### 2.1.2 Longitudinal cohort

We tested the findings in the cross-sectional cohort by analyzing changes over time in HOMA-β using repeat samples from individuals. The repeated samples were recruited from our own cohort and an external cohort from the same geographic region (Zhengzhou City, China). Both cohorts used the same inclusion and exclusion criteria for T2DM.

In our own cohort, repeat HOMA-β measurements were available for 1213 subjects from the 2898 individuals in the cross-sectional cohort. After excluding subjects whose HOMA-β measurement time points fell into different stage intervals (referring to the three stages described in the ‘Results’ section), a total of 3316 HOMA-β values from 1137 patients were included in the final analysis. A median (IQR) of 2 (2, 3) results were available per subject, over a median (IQR) of 3.5 (1.8, 5.9) years of follow-up.

The external data come from a prospective cohort based at the National Metabolic Management Center (MMC) (16) sub-center established by the Fifth Affiliated Hospital of Zhengzhou University. This study was approved by the Ethics Committee of the Fifth Affiliated Hospital of Zhengzhou University (No. KY20211019), and all patients signed the informed consent form for enrollment. A total of 705 HOMA-β measurements from 237 patients were included in the analysis. A median (IQR) of 2 (2, 3) results were available per patient, over a median (IQR) of 9.2 (3.3, 25.2) months of follow-up.

The following information was collected at each visit: disease duration, BMI, systolic blood pressure (SBP), and diastolic blood pressure (DBP). Moreover, serum was collected for measurements of plasma glucose, HbA1c, C-peptide, triglycerides (TG), low-density lipoprotein cholesterol (LDL-C), uric acid (UA), and diabetes-associated autoantibodies.

### 2.2 Laboratory analysis

Height, weight, and blood pressure were measured with a standardized procedure, and BMI was calculated. Levels of FPG, TG, LDL-C, and UA were determined using the automatic chemistry system at the core laboratory of the Second Affiliated Hospital of Zhengzhou University. HbA1c was measured using high-performance automated liquid chromatography (HLC-723G8, Tosoh, Japan). GADA and ICA were qualitatively detected using a standard immunoblot kit (BLOT, Shenzhen, China). Plasma C-peptide was tested using the electrochemical luminescence method (cobas-E411, Basel, Switzerland). Furthermore, the inter-assay and intra-assay variation coefficients were 3.7–4.1% and 1.0–3.3%, respectively. The lower detection limit of the assay was 16.7 pmol/L.

Fasting plasma glucose (FPG) and fasting C-peptide (FCP) were tested in patients fasting for at least 8 hours. HOMA-β was calculated using the following formula: HOMA-β (%) =0.27×FCP (pmol/L)/[FPG (mmol/L)-3.5] (17).

Metabolic Syndrome (MetS) was defined using the 2017 Chinese Diabetes Society’s (CDS) criteria (18) and was diagnosed when three or more of the following criteria were met: (a) abdominal obesity: waist circumference ≥90 cm in men and ≥85 cm in women; (b) hyperglycemia: FBS ≥6.1 mmol/L or 2-hour PBS ≥7.8 mmol/L or previously diagnosed diabetes with treatment; (c) hypertension: blood pressure ≥130/85 mmHg or currently under antihypertension therapy; (d) fasting TG ≥1.70 mmol/L; (e) fasting HDL-C<1.04 mmol/L.

### 2.3 Statistical analysis

HOMA-β results were natural log transformed for analysis as the distribution of its values was heavily skewed. Initial analysis of cross-sectional data used non-linear regression modeling to examine the association between duration and HOMA-β. Generalized additive models (GAM) were used to explore the initial shape of the associations. Notably, this revealed a pattern consistent with three phases that could be modeled with three lines of best fit. Segmented regression was then used to determine the optimal breakpoints where the lines of best fit would meet and to enable calculation of the intercept and slopes of different phases, thereby modeling the starting point and rate of HOMA-β decline. The intercepts were back-transformed (using the exponential) to estimate HOMA-β levels at diagnosis from the models. As slopes were on a log scale, they were interpreted as percentage change per year (calculated from the exponential of the β coefficient minus 1).

For the longitudinal analysis, data were split into three groups for the three phases: before and after the optimal breakpoints identified from cross-sectional analysis. The slopes of the three phases were determined using mixed effects models to model HOMA-β against duration, with random effects at the patient level to allow each patient to contribute multiple values at different time points. We used a random-intercept, random-slope model to allow for variability between individuals. Moreover, we excluded those whose first value was below the lower limit of detection of the assay to ensure the finding did not represent a floor effect (i.e., that the results were not an artifact of those below the lower limit of the assay unable to fall).

We further explored clinical indicators related to residual HOMA-β levels using cross-sectional data. Based on longitudinal data, we incorporated the duration of diabetes as a continuous time-varying covariate in the mixed effect model. We explored significant associations (statistical interactions) between determinants of interest and duration of diabetes on HOMA-β levels.

All analyses were carried out in SPSS version 24.0 or R version 3.2.2, including the mgcv package (for generalized additive models), lme4 package (for mixed effects models), and segmented package (for segmented regression). Normally distributed data were presented as mean ± SD. Variables with a skewed distribution were reported as median (quartile range: 25th, 75th). Categorical variables were expressed as percentages. Normally distributed data were compared using ANOVA, and non-normally distributed data were compared using the Wilcoxon Rank Sum and Spearman correlation test. Categorical variables were compared using a chi-squared test. Furthermore, we performed univariate analyses to compare clinical features in subjects with different levels of residual beta-cell function. Multivariate logistic regression analyses were performed to further investigate the possible determinants of beta-cell function preservation. Two-sided statistical tests were performed, and a p-value <0.05 (two-sided) was considered statistically significant.

## 3 Results

### 3.1 Baseline clinical features

Clinical and biochemical characteristics of the 2898 patients in cross-sectional analysis are presented in **Table 1**. At baseline, the mean age was 60.0 years old (ranging from 12.0 to 94.0), and 55.7% were male. The mean age of diagnosis was 48.4 years old (ranging from 7.1 to 86.5), and the mean disease duration was 8.4 years (ranging from 0.1 to 50.0). The average BMI (IQR) level was 25.0 (23.0, 27.4) kg/m^2^, meeting the diagnostic criteria for overweight in China (19). The average HbA1c (IQR) level was 61.7 (50.8, 81.4) mmol/mol. Moreover, the subjects’ blood pressure, glucose, and lipids levels did not meet the strict control targets, and almost 80% of the subjects combined at least one type of diabetic microvascular or neurological complications.

**Table 1:** Baseline clinical features of 2898 subjects.

Variable	Value
FCP, pmol/L	865.8 (599.4, 1185.5)
HOMA-β, %	58.0 (35.9, 95.6)
Age, year	60.0 (50.0, 69.0)
Age of diagnosis, year	48.4 (40.3, 56.7)
Duration, year	8.4 (2.6, 15.8)
Male, n (%)	1614 (55.7)
BMI, kg/m^2^	25.0 (23.0, 27.4)
HbA1c, mmol/mol	61.7 (50.8, 81.4)
HbA1c, %	7.8 (6.8, 9.6)
LDL-C, mmol/L	2.73 (2.07, 3.45)
TG, mmol/L	1.53 (1.07, 2.34)
UA, mmol/L	308.0 (250.0, 373.0)
SBP, mmHg	130.0 (125.0, 138.0)
DBP, mmHg	78.0 (74.0, 85.0)
Smoking, n (%)	573(19.8)
Drinking, n (%)	292 (10.1)
Diabetic retinopathy, n (%)	489 (16.9)
Diabetic nephropathy, n (%)	591 (20.4)
Diabetic neuropathy, n (%)	2286 (78.9)
Insulin, n (%)	923 (31.8)
Metformin, n (%)	1970 (68.0)
Sulfonylureas, n (%)	551 (19.0)
TZDs, n (%)	191 (6.6)
α-glucosidase inhibitors, n (%)	2086 (72.0)
SGLT-2 inhibitors, n (%)	1101 (38.0)
GLP-1 Ras, n (%)	397 (13.7)
DPP-4 inhibitors, n (%)	548 (18.9)
MetS, n (%)	1944 (67.1)

Data are presented as mean ± SD, median (25th, 75th) or n (%), depending on variable type and distribution. BMI, body mass index; TG, total cholesterol; LDL-C, low density lipoprotein cholesterol; UA, urea; SBP, systolic blood pressure; DBP, diastolic blood pressure.

### 3.2 Cross-sectional analysis identified multi-phases of beta-cell function change

**Figure 2:**
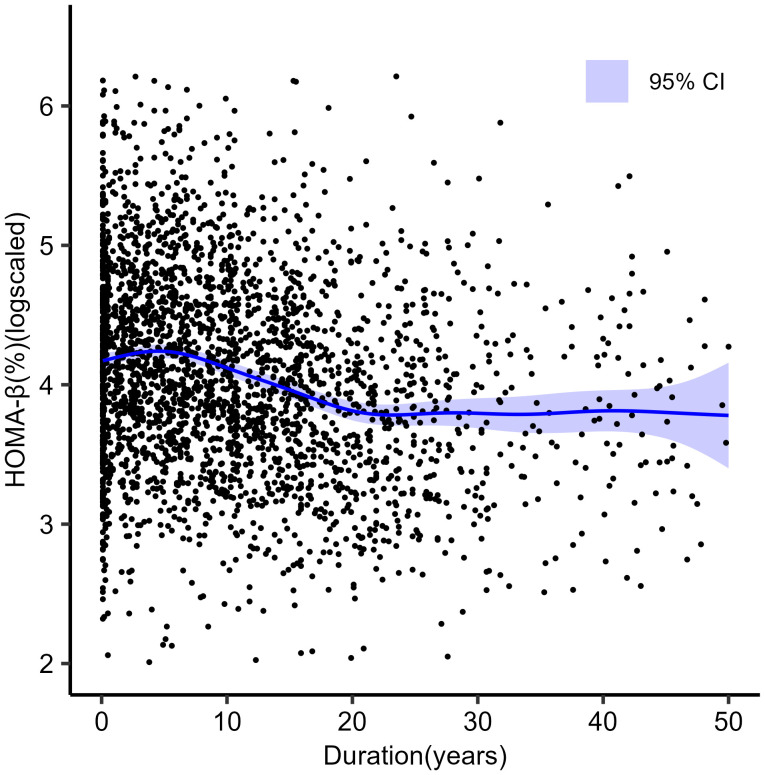
Scatterplots of ln (HOMA-β) against duration of diabetes in 2898 individuals with type 2 diabetes. The blue line shows generalized additive modelling (non-linear) line of best fit.

We used GAM to investigate the changing pattern of HOMA-β with the course of the disease. HOMA-β was found to have a nonlinear association with the disease duration, suggesting three phases. As shown in **Figure 2**, the first phase lay within about 4 years from diagnosis, during which the curve rose slightly. This was followed by a second phase up until around 21 years after diagnosis, during which the curve declined significantly, and the HOMA-β level experienced a substantial reduction. Then, it progressed to the third phase, where the curve flattened, indicating a continuous low level of HOMA-β.

**Figure 3:**
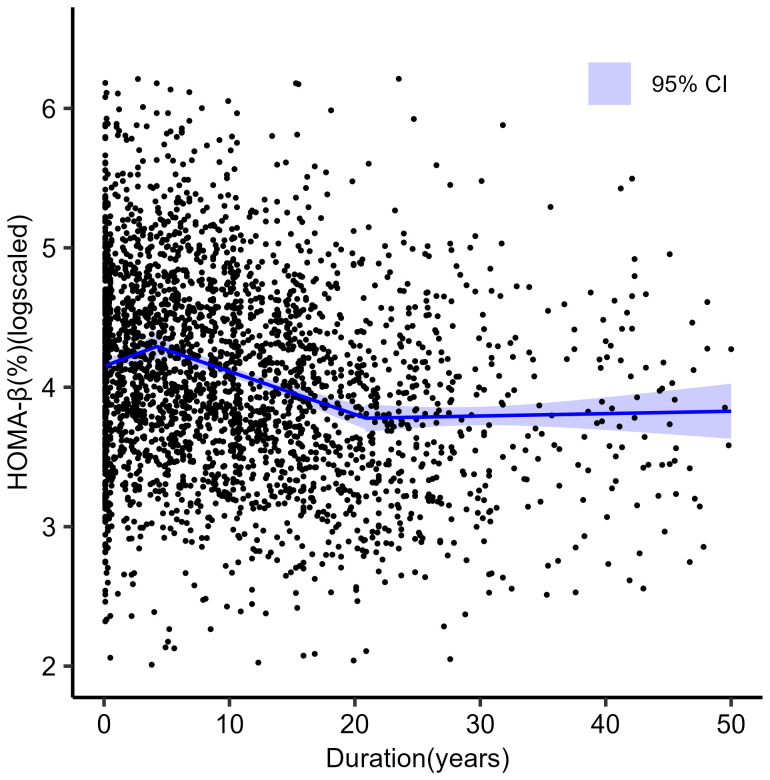
Scatterplots of ln (HOMA-β) against duration of diabetes in 2898 individuals with type 2 diabetes. The blue line shows three straight lines of best fit meeting at the optimal breakpoint from segmented regression analysis.

Segmented regression was used to model the slopes of different phases. The estimated HOMA-β level at diagnosis was 63.4% (95%CI: 61.4, 65.4). The breakpoints at which the slope changed were modeled at 4.20 (95%CI: 2.31, 6.09) years and 20.90 (95%CI: 16.93, 24.84) years from diagnosis. During the first stage, the annual change of HOMA-β was 3.34% (95% CI: 0.04, 6.52), suggesting that beta-cell function would experience a brief ‘ascending phase’ after clinical diagnosis. During the second stage, the HOMA-β decayed at -3.04% (95% CI: -3.78, -2.29) per year, forming the main ‘decline phase’ of beta-cell function. Then, during the third stage, the annual change for HOMA-β descended to 0.17% (95% CI: -0.72, 1.05) per year, suggesting a ‘plateau phase’ thereafter. **Figure 3** shows the fitted slopes and **Table 2** shows the estimated parameters during the three phases.

**Table 2:** Estimated levels and decline rates of HOMA-β from segmented regression analysis of cross-section data.

Phase	Parameter
Phase 1	
Estimated HOMA-β (%) level at diagnosis ^§^ [95% CI]	63.4 [61.4, 65.4]
Slope 1 [95% CI]	0.0339 [0.0004, 0.0674] ***
Percentage change per year ^†^ [95% CI]	3.34% [0.04, 6.52] ***
Phase 2	
Breakpoint1 (year) [95% CI]	4.20 [2.31, 6.09]
Slope 2 [95% CI]	-0.0309 [-0.0385, -0.0232] ***
Percentage change per year ^†^ [95% CI]	-3.04% [-3.78, -2.29] ***
Phase 3	
Breakpoint2 (year) [95% CI]	20.90 [16.93, 24.84]
Slope 3 [95% CI]	0.0017 [-0.0073, 0.0106]
Percentage change per year ^†^ [95% CI]	0.17% [-0.72, 1.05]

^§^Exponential of intercept taken to show estimated HOMA-β at diagnosis; ^†^calculated from the exponential of β (the regression slope)-1; ***p<0.001.

### 3.3 Longitudinal data validated the multi-phases in the cross-sectional model

The cross-sectional model was verified using longitudinal data from both internal and external cohorts. In line with the estimated inflection breakpoints, we calculated the slopes of each phase in the HOMA-β curve with the course of the disease using the longitudinal data. The numbers of individuals, observations, and the estimated slopes at distinct phases are presented in **Table 3**. The decline pattern of HOMA-β was similar to that seen in the cross-sectional model. It consisted of an initial ‘ascending stage,’ followed by a ‘decline stage’ with substantial fall, and then a continuous ‘plateau stage’.

**Table 3:** Estimated levels and decline rates of HOMA-β from mixed effect models of longitudinal data.

Phase	Internal cohort	External cohort
Phase 1: 0-4.2 years from diagnosis		
Estimated HOMA-β (%) level at diagnosis § [95% CI]	62.7 [55.2, 71.2]	56.7 [44.3, 68.8]
Number of individuals/observations	177/403	62/142
Slope 1[95% CI]	0.0402[-0.00837, 0.0888] ***	0.1109[0.0082, 0.2136] *
Percentage change per year † [95% CI]	3.94[-0.01, 8.49] ***	11.73[0.82, 23.89] *
Phase 2: 4.3-20.9 years from diagnosis		
Number of individuals/observations	688/2081	117/411
Slope 2[95% CI]	-0.0398[-0.0492, -0.0304] ***	-0.0291[-0.0520, -0.0059] *
Percentage change per year † [95% CI]	-4.06[-5.04, -3.09] ***	-2.87[-5.07, -0.59] *
Phase 3: 21.0-50.0 years from diagnosis		
Number of individuals/observations	272/832	58/152
Slope 3[95% CI]	-0.0008[-0.0122, 0.0106]	-0.0030 [-0.0061, 0.0001]
Percentage change per year † [95% CI]	-0.08[-1.22, 1.05]	-0.03[-0.06, 0.01]

^§^Exponential of intercept taken to show estimated HOMA-β at diagnosis;^†^calculated from the exponential of β(the regression slope)-1; * p<0.05; ***p<0.001.

### 3.4 Factors associated with residual beta-cell function

The 2898 subjects in the cross-sectional analysis were grouped according to their residual HOMA-β levels. Subjects with their HOMA-β points above the GAM model fitting curve (**Figure 1**) were considered to have ‘preferable residual beta-cell function’ and classified into ‘Group 1’. In contrast, others having ‘inferior beta-cell function’ were assigned to ‘Group 2’.

Univariate analysis showed that compared to Group 2, age (61.0 vs. 58.0 years, p<0.001), age of diagnosis (49.3 vs. 47.3 years, p<0.001), BMI (25.7 vs. 24.5 kg/m², p<0.001), levels of LDL-C (2.85 vs. 2.57 mmol/L, p<0.001), UA (320.0 vs. 294.0 mmol/L, p<0.001) and proportion of MetS (71.5% vs. 62.8%, p<0.001) were higher in Group 1. In comparison, the HbA1c level (55.2 vs.71.6 mmol/mol, p<0.001) and proportion of insulin use (28.3% vs. 35.4%) were lower in Group 1. Notably, the proportions of diabetic retinopathy (19.2% vs. 14.6%), diabetic neuropathy (82.5% vs. 75.5%) and in Group 1 were significantly higher than those in Group 2 (**Table 4**).

**Table 4:** Clinical characteristics of subjects with distinct levels of residual β-cell function.

Variable	Group 1 (n=1433)	Group 2 (n=1465)	p value
FCP, pmol/L	1082.4 (819.2, 1418.6)	682.7 (506.2, 905.8)	0.000***
HOMA-β, %	96.50 (73.70, 137.00)	36.23 (26.05, 46.53)	0.000***
Age, year	61.0 (51.0, 70.0)	58.0 (49.0, 68.0)	0.000***
Age of diagnosis, year	49.3 (41.3, 57.7)	47.3 (39.2, 55.6)	0.000***
Duration, year	8.4 (2.6, 15.8)	8.2 (2.5, 15.7)	0.735
Male, n (%)	796 (55.4)	818 (55.8)	0.876
BMI, kg/m^2^	25.7 (23.4, 27.8)	24.5 (22.6, 26.8)	0.000***
HbA1c, %	7.2 (6.4, 8.6)	8.7 (7.4, 10.4)	0.000***
HbA1c, mmol/mol	55.2 (46.4, 70.5)	71.6 (57.4, 90.2)	0.000***
SBP, mmHg	130.0(125.0,139.0)	130.0(125.0,138.0)	0.541
DBP, mmHg	78.0(73.0, 84.0)	78.0(74.0,85.0)	0.141
Smoking, n (%)	281(19.6)	292 (19.9)	0.827
Drinking, n (%)	144 (10.0)	148 (10.1)	0.962
LDL-c, mmol/L	2.85 (2.20, 3.52)	2.57 (1.96, 3.37)	0.000***
TG, mmol/L	1.55 (1.09, 2.33)	1.50 (1.04, 2.35)	0.196
UA, mmol/L	320.0 (262.0, 386.3)	294.0 (240.0, 358.8)	0.000***
Diabetic retinopathy, n (%)	208 (14.6)	281 (19.2)	0.001**
Diabetic nephropathy, n (%)	305 (21.3)	286 (19.5)	0.239
Diabetic neuropathy, n (%)	1080 (75.5)	1206 (82.5)	0.000***
Insulin, n (%)	405 (28.3)	518 (35.4)	0.000***
Metformin, n (%)	960 (70.0)	1010 (68.9)	0.290
Sulfonylureas, n (%)	301 (21.0)	250 (17.1)	0.063
TZDs, n (%)	106 (7.4)	85 (5.8)	0.135
α-glucosidase inhibitors, n (%)	1050 (73.3)	1036 (70.7)	0.142
SGLT-2 inhibitors, n (%)	581 (40.5)	520 (35.5)	0.078
GLP-1 Ras, n (%)	216 (15.1)	181 (12.4)	0.089
DPP-4 inhibitors, n (%)	286 (20.0)	262 (17.9)	0.115
MetS, n (%)	1024 (71.5)	920 (62.8)	0.000***

Data are presented as mean ± SD, median (25th, 75th) or % (n), depending on variable type and distribution. BMI, body mass index; TG, total cholesterol; LDL-C, low density lipoprotein cholesterol; UA, urea; SBP, systolic blood pressure; DBP, diastolic blood pressure. **p<0.01;***p<0.001.

Further multivariate analyses showed that higher BMI levels (OR = 1.103, [95%CI 1.048, 1.161], p<0.001), UA levels (OR = 1.003, [95%CI 1.001, 1.005], p=0.014), MetS (OR = 1.526, [95%CI 1.021, 2.279], p=0.039) and lower HbA1c levels (OR = 0.696, [95%CI 0.628, 0.771], p<0.001) were independently associated with higher residual HOMA-β level (**Table 5**).

**Table 5:** Possible determinants of residual β-cell function.

Variable	OR	95% CI	p value
Age, year	0.987	0.967-1.009	0.241
Age of diagnosis, year	1.012	0.989-1.036	0.317
BMI, kg/m^2^	1.103	1.047-1.161	0.000***
HbA1c, mmol/mol	0.695	0.627-0.771	0.000***
LDL-C, mmol/L	0.891	0.735-1.079	0.238
UA, mmol/L	1.003	1.001-1.005	0.015*
Diabetic retinopathy	0.997	0.648-1.513	0.989
Diabetic neuropathy	0.693	0.429-1.119	0.134
Insulin use	0.929	0.623-1.386	0.719
MetS	1.526	1.021-2.279	0.039*

Multivariable logistic regression including all the variables with significant differences in the univariant analysis was performed. *p<0.05; ***p<0.001. BMI, body mass index; LDL-C, low density lipoprotein cholesterol; UA, urea.

### 3.5 Factors associated with beta-cell function decline rate

We focused on the second stage in the above models, which was the main ‘decline phase’ of HOMA-β. Using longitudinal follow-up data, the mixed effect linear model showed that HOMA-β levels decreased with increased disease duration and HbA1c levels. Notably, the age of diagnosis was associated with HOMA-β decline rate since there was a significant interaction (Coefficient=0.0009 [95%CI: 0.0002, 0.0016]) between age of diagnosis and disease duration on HOMA-β levels over time. This suggests that the earlier the disease diagnosis, the faster the rate at which HOMA-β declines with the disease course (**Table 6**).

**Table 6:** Longitudinal mixed model exploring the effect of clinical characteristics on FCP and HOMA-β levels over time.

Variable	HOMA-β	
	β [95% CI]	p value
Duration, year	-0.0874 [-0.1459, 0.0291]	0.003**
Male	-0.0320 [-0.2393, 0.1753]	0.762
Age of diagnosis, year	-0.0029 [-0.0121, 0.0064]	0.546
BMI (kg/m^2^)>25.0	-0.0036 [-0.2372, 0.2302]	0.976
HbA1c, mmol/mol	-0.0648 [-0.1227, -0.0068]	0.028*
UA, mmol/L	0.0003 [-0.0007, 0.0012]	0.591
Insulin use	-0.0016 [-0.3203, 0.2878]	0.916
MetS	-0.1638 [-0.4560, 0.1284]	0.271
Duration: Male	0.0012 [-0.0142, 0.0166]	0.880
Duration: Age of diagnosis	0.0009 [0.0002, 0.0016]	0.012*
Duration: BMI	-0.0063[-0.0237, 0.0111]	0.477
Duration: HbA1c	0.0013[-0.0029, 0.0056]	0.539
Duration: UA	0.0001 [-0.0001, 0.0001]	0.620
Duration: Insulin use	0.0079 [-0.153, -0.0312]	0.503
Duration: MetS	0.0179 [-0.0046, 0.0404]	0.119

This model shows that age of diagnosis is associated with HOMA-β decline over time, because there was a significant interaction between age of diagnosis and DM duration on ln(FCP) and ln(HOMA-β) levels; BMI, body mass index; UA: urea; *p<0.05; **p<0.01.

## 4 Discussion

The “three-phase HOMA-β model” is clinically relevant and highly consistent with previous literature. Firstly, the “ascending phase” usually emerges after short-term interventions (such as intensive insulin therapy, oral medications, or lifestyle interventions), accompanied by increased insulin secretion and improved glycemic control. This so-called “clinical remission period” (9, 20–22) is equivalent to the first stage in this study. Secondly, the following ‘decline phase’ was also witnessed. The well-known UKPDS reported a decline rate of 5% per year in British type 2 diabetic patients (2), while in Chinese populations, this figure was 2% (11). Different genetic backgrounds, intervention programs may contribute to the varied decline rates. Thirdly, as for the ‘plateau phase,’ the Veterans Affairs Diabetes Trial (VADT) showed that the C-peptide levels decreased progressively from 0–3 years after diagnosis until 15 years’ duration and then remained stable after 16–18 years, suggesting that beta-cell function loss would tend to stabilize at a certain point (10).

Moreover, the continues change pattern was also observed. The Belfast Diet Study (8) used HOMA-β to explore changes in beta-cell function in type 2 diabetic patients and found an initial slow decline (1.7% decline per year) period followed by a rapid decline (18.2% decline per year). Unfortunately, the follow-up was terminated before the 10th year post-diagnosis, so no further changes thereafter were observed. More consistent results came from another Chinese study (23). Ding et al. followed 1570 type 2 diabetic subjects for up to 35 years. They found that beta-cell function remained unchanged within 5 years after diagnosis, declined by 2% annually between 5 and 22 years, and remained at a low level thereafter (23). Despite the detailed differences, the above results are consistent with this study.

As for the potential underlying pathophysiological mechanisms, each phase needs to be discussed separately. The beta-cell function decline in type 2 diabetes broadly resulted from beta-cell number reduction, beta-cell exhaustion, and beta-cell de-differentiation or trans-differentiation into other cell types (24), all closely related to glucotoxicity/lip-toxicity (25). Apart from beta-cell number reductions, which are difficult to reverse, the other two processes can be reversed after removing glucotoxicity/lip-toxicity (24).

**Figure 4:**
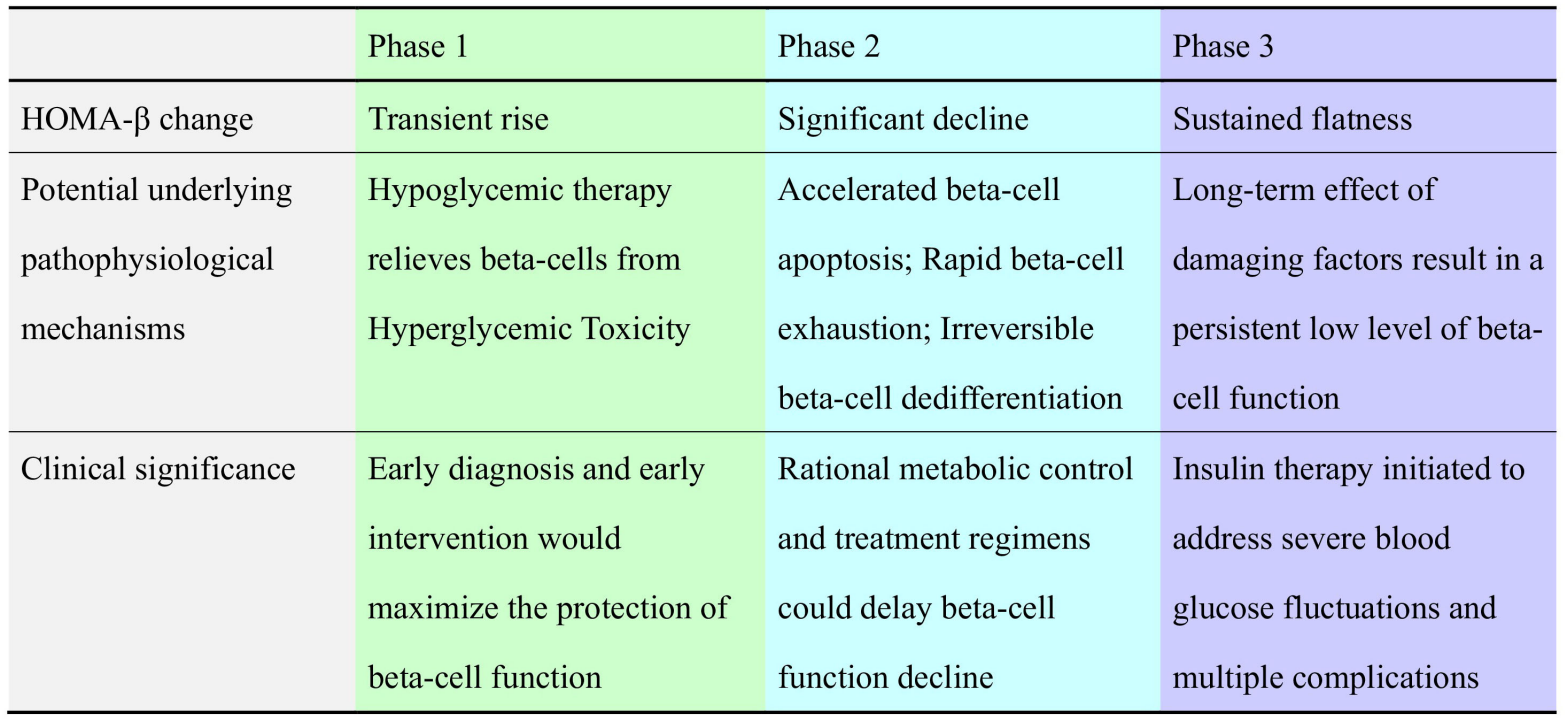
Potential underlying pathophysiological mechanisms and clinical features of the ‘three-phase model’.

In this model, the first ‘ascending phase’ may result from the glucose and lipid-lowering therapy initiated after diagnosis which improved the underlying pathophysiological conditions and restored beta-cell function. Therefore, early screening and treatment should be conducted to seize the time window for beta-cell protection (26) (**Figure 4**). The following ‘decline phase’ was described by A. Bagust as the ‘fully developed stage of type 2 diabetes’, which was characterized by a significant irreversible decline of beta-cell function (8). Its relevant pathophysiological processes may include accelerated beta-cell apoptosis, rapid beta-cell exhaustion, and irreversible beta-cell dedifferentiation (8) (**Figure 4**). Moreover, the Counterpoint Study (27) and The Scandinavian Obesity Study (28) confirmed that as the duration of diabetes increases, an irreversible ‘turning point’ would be passed with beta-cells underwent irreversible damage. Regarding this ‘turning point’, B. TOPP et al. put forward a hypothesis (29):a regulation system would attempt to maintain the insulin secretion level by reducing beta-cell loss and/or increasing beta-cell replication at the early stage of the disease. However, the regulatory ability gradually weakens and eventually reaches an unstable saddle point at which even a modest event (such as an infection or an over-indulgence) would propel the subject into the subsequent phase of sudden accelerated disease progression. During the last ‘plateau phase’, patients tend to have persistently low levels of beta-cell function, resulting in failed oral drug treatment, drastic blood sugar fluctuation, and multiple complications (3–6). Insulin therapy often needs to be initiated at this stage (**Figure 4**).

We further analyzed clinical factors associated with residual beta-cell function. Univariate analysis showed inverse correlations of diabetic retinopathy and diabetic neuropathy, which was consistent with previous reports (6). After adjusting for multiple variables, higher BMI, UA and MetS proportion levels were still correlated with higher residual HOMA-β level. The positive correlations were particularly significant among overweight or obese subjects (30, 31). Notably, this did not mean MetS is beneficial to beta-cell preservation, since free fatty acids, UA, and related inflammation could cause insulin resistance and a compensatory increase in insulin secretion, which would ultimately damage beta-cell function (32, 33). In this study, the average BMI level was 25.0 kg/m², reaching the criteria of overweight for Chinese (16). Therefore, fat loss and muscle building should be conducted in this group of people (34). The elevated HbA1c and reduced beta-cell function were thought to be causally related: the glucotoxicity may damage beta-cell function (24) while correspondingly, the decreased insulin level would cause an increased blood glucose level (5). Therefore, active blood glucose control can break this vicious cycle and prevent disease progression.

We also observed that insulin use was related to lower residual HOMA-β levels. However, prior studies demonstrated that early insulin intervention preserves beta-cell function (35). This suggests that insulin treatment would be initiated belatedly in our patients, potentially missing the optimal window for beta-cell protection. Thus, in clinical practice, adequate attention should be paid to beta-cell function protection. Timely use of agents with potential β-cell-protective effects [e.g., GLP-1Ras (36), SGLT-2 inhibitors (37), DPP-4 inhibitors (38)] is advisable.

Moreover, we found that early disease onset was associated with faster beta-cell function decline. Similar results have been reported in the ‘Restoring Insulin Secretion (RISE) Study’ and ‘Treatment Options for Type 2 Diabetes in Adolescents and Youth (TODAY) Study’ (39, 40). This arises from both genetic and environmental factors. A genome-wide association study (GWAS) by the Progress in Diabetes Genetics in Youth Consortium (ProDiGY) (41) highlighted the key role of genetic background. In contrast, socio-environmental factors—such as later bedtimes (42) and fewer medical visits (43)—may reduce residual beta-cell function. Unfortunately, the incidence and prevalence of early-onset type 2 diabetes (in those aged ≤40 years) are rising globally (1). Thus, early combination therapy is recommended for young adults (<40 years) with type 2 diabetes (44).

The strengths of our study are as follows. First, we had a large sample size of 2892 subjects and a prolonged disease course coverage of 50 years. This ensured the statistical power and enabled long-term continuous observation. Second, the diabetic-associated antibodies including IAA, GADA and ICA, were detected to exclude autoimmune diabetes such as LADA (45). This largely reduces the heterogeneity of the cohort. Third, this study combined cross-sectional and longitudinal data from multi-centers, which enhanced the reliability of the conclusions.

The limitations of our study are as follows. First, although the results are consistent with previous studies, it must be noted that the ‘three-phase HOMA-β model’ is still largely based on statistical analysis of a single Chinese Han population. The turning points and change rates within each phase may vary among different populations. Also, variability in treatment regimens, survivor bias, or misclassification of disease onset might affect the existence of multi-phases. Therefore, multi-center, large-sample prospective studies across diverse ethnic groups should be conducted in the future, and the underlying pathophysiological mechanisms also require further investigations. Second, despite the strict control over the inclusion and exclusion criteria, it is still possible that some LADA and classic T1DM patients were included, which increases the heterogeneity of the cohort. Third, this study used HOMA-β to evaluate beta-cell function, which reflected basal insulin secretion (46) but failed to capture the dynamic changes in insulin response. We will strive to adopt OGTT-derived indices in future studies to more comprehensively reflect beta-cell function.

In conclusion, this study revealed the complex model of beta-cell function change in type 2 diabetes. Based on this model, the pathophysiological mechanisms underlying different phases would be investigated and targeted clinical interventions could be implemented in the specific population.

## Data Availability

The original contributions presented in the study are included in the article/Supplementary Material. Further inquiries can be directed to the corresponding authors.
