# Supplementary material for: Multi-phases of islet beta-cell function change in type 2 diabetes mellitus and its influencing factors
Source: Front Endocrinol (Lausanne). 2025 Oct 10;16:1602796. doi: 10.3389/fendo.2025.1602796 (PMC12549277; doi:10.3389/fendo.2025.1602796)
Supplement: Supplementary file 1 [file DataSheet1.docx]

S**ubgroup analyses for medication class and baseline metabolic control were conducted according to suggestions from Reviewer 1**

1. We divided 2,898 patients into groups by gender and observed females had higher HOMA-β levels at initial diagnosis than males (54.13% vs 51.89%). There were no statistically significant differences in the inflection points or decline rates at each stage between the two groups (Supplementary Table 1, Supplementary Figure 1).

Supplementary Table 1. HOMA-β change patterns in males and females

|  | Female  (n=1206) | Male  (n=1692) | P |
| --- | --- | --- | --- |
| Phase 1 |  |  |  |
| HOMA-β at diagnosis（%） [95% CI] ^§^ | 54.13 [54.00, 54.26] | 51.89 [51.79, 51.99] | 0.000* |
| β1 [95% CI] | 0.0351 [-0.0247, 0.0950] | 0.0295 [-0.0056, 0.0647] | 0.823 |
| HOMA-β change per year（%）[95% CI]^†^ | 3.56 [-2.44, 10.00] | 3.00 [-0.56, 6.69] | 0.823 |
| Phase 2 |  |  |  |
| Break point（year）[95% CI] | 3.74 [0.82, 6.67] | 4.80 [2.34, 7.26] | 0.398 |
| β2 [95% CI] | -0.0297 [-0.0419, -0.0176] | -0.0323 [-0.0428, -0.0218] | 0.683 |
| HOMA-β change per year（%）[95% CI] ^†^ | -2.93 [-4.10, -1.74] | -3.18 [-4.19, -2.16] | 0.682 |
| Phase 3 |  |  |  |
| Break point（year）[95% CI] | 20.10 [13.97, 26.23] | 21.35 [16.26, 26.45] | 0.578 |
| β3 [95% CI] | 0.0011 [-0.0107, 0.0129] | 0.0019 [-0.0112, 0.0150] | 0.847 |
| HOMA-β change per year（%）[95% CI] ^†^ | 0.11 [-1.06, 1.30] | 0.19 [-1.11, 1.51] | 0.845 |

Note:§exponential of intercept taken to show estimated HOMA-β at diagnosis; †calculated from the exponential of β (the regression slope) -1;***p＜0.001.


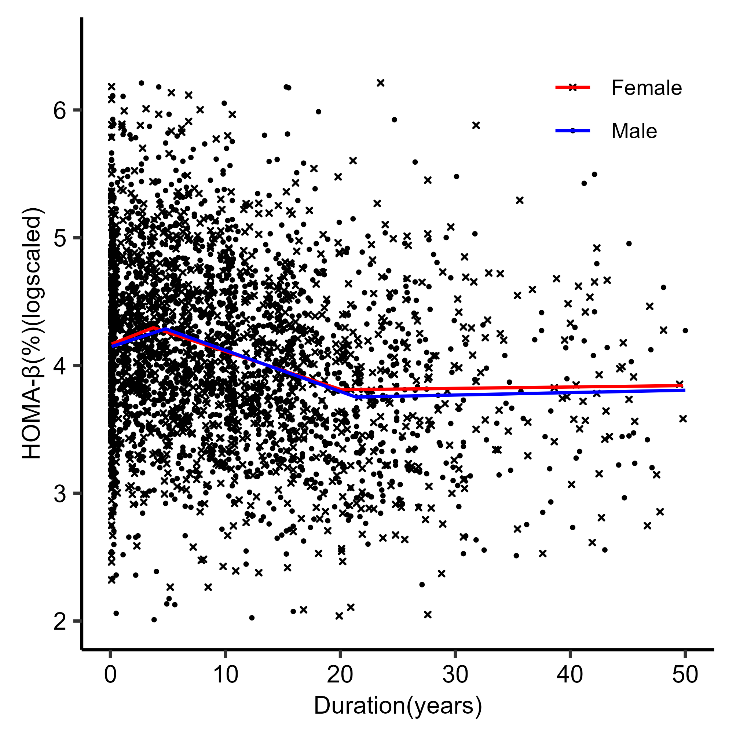


Supplementary Figure 1. HOMA-β change in males and females

2. We divided 2,898 patients according to their diagnosis age (above or below the median diagnosis age). We observed patients diagnosed late had higher HOMA-β levels at initial diagnosis than those diagnosed early (68.23% vs 58.56%). There were no statistically significant differences in the inflection points or decline rates at each stage between the two groups (Supplementary Table 2, Supplementary Figure 2).

Supplementary Table 2. HOMA-β change patterns in early or late onset patients

|  | Early onset  (n=1441) | Late onset  (n=1457) | P |
| --- | --- | --- | --- |
| Phase 1 |  |  |  |
| HOMA-β at diagnosis（%） [95% CI] ^§^ | 58.56 [59.00, 59.29] | 68.23 [61.56, 74.44] | 0.003* |
| β1 [95% CI] | 0.0213 [-0.0167, 0.0630] | 0.0415[-0.0143, 0.0972] | 0.542 |
| HOMA-β change per year（%）[95% CI]^†^ | 2.15 [-1.66, 6.50] | 4.24 [-1.42, 10.21] | 0.562 |
| Phase 2 |  |  |  |
| Break point（year）[95% CI] | 5.10 [1.44, 8.76] | 3.52 [1.28, 5.76] | 0.471 |
| β2 [95% CI] | -0.0279 [-0.0416, -0.0142] | -0.0324 [-0.0417, -0.0231] | 0.506 |
| HOMA-β change per year（%）[95% CI] ^†^ | -2.75 [-4.06, -1.41] | -3.19 [-4.07, -2.28] | 0.504 |
| Phase 3 |  |  |  |
| Break point（year）[95% CI] | 20.15 [14.33, 25.97] | 21.30 [13.82, 28.78] | 0.711 |
| β3 [95% CI] | 0.0022 [-0.0074, 0.0117] | -0.0030 [-0.0309, 0.0248] | 0.795 |
| HOMA-β change per year（%）[95% CI] ^†^ | 0.22 [-0.74, 1.18] | -0.30 [-3.04, 2.51] | 0.787 |

Note:§exponential of intercept taken to show estimated HOMA-β at diagnosis; †calculated from the exponential of β (the regression slope) -1;***p＜0.001.


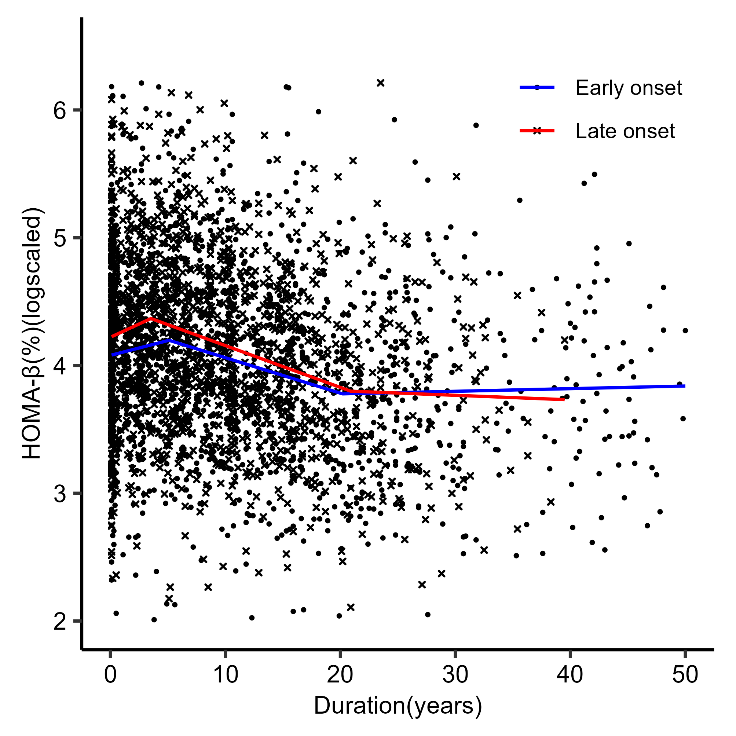


Supplementary Figure 2. HOMA-β change in early or late onset patients

3. We divided 2,898 patients according to their BMI levels (above or below the median BMI level). We observed patients with higher BMI had higher HOMA-β levels at initial diagnosis than those with lower BMI (62.15% vs 61.94%). There were no statistically significant differences in the inflection points or decline rates at each stage between the two groups (Supplementary Table 3, Supplementary Figure 3).

Supplementary Table 3. HOMA-β change patterns in patients with high or low levels of BMI

|  | Low BMI  (n=1441) | High BMI  (n=1457) | P |
| --- | --- | --- | --- |
| Phase 1 |  |  |  |
| HOMA-β at diagnosis（%） [95% CI] ^§^ | 61.94 [61.76, 62.13] | 62.15 [62.00, 62.30] | 0.002* |
| β1 [95% CI] | 0.0244 [-0.0136, 0.0623] | 0.0090 [-0.0421, 0.0198] | 0.568 |
| HOMA-β change per year（%）[95% CI]^†^ | 2.47 [-1.36, 0.64] | 0.90 [-0.41, 0.20] | 0.227 |
| Phase 2 |  |  |  |
| Break point（year）[95% CI] | 5.30 [2.27,8.33] | 6.20 [2.70, 9.70] | 0.746 |
| β2 [95% CI] | -0.0352 [-0.0489, -0.0215] | -0.0309 [-0.0421, -0.0198] | 0.674 |
| HOMA-β change per year（%）[95% CI] ^†^ | -3.45 [-4.75, -2.12] | -3.04 [-4.10, -0.20] | 0.815 |
| Phase 3 |  |  |  |
| Break point（year）[95% CI] | 19.91 [15.23, 24.49] | 21.54 [16.11, 26.97] | 0.658 |
| β3 [95% CI] | 0.0053 [-0.0052, 0.0159] | -0.0003 [-0.0111, 0.0104] | 0.657 |
| HOMA-β change per year（%）[95% CI] ^†^ | 0.53 [-0.52, 1.59] | -0.03 [-1.11, 1.04] | 0.657 |

Note:§exponential of intercept taken to show estimated HOMA-β at diagnosis; †calculated from the exponential of β (the regression slope) -1;***p＜0.001.


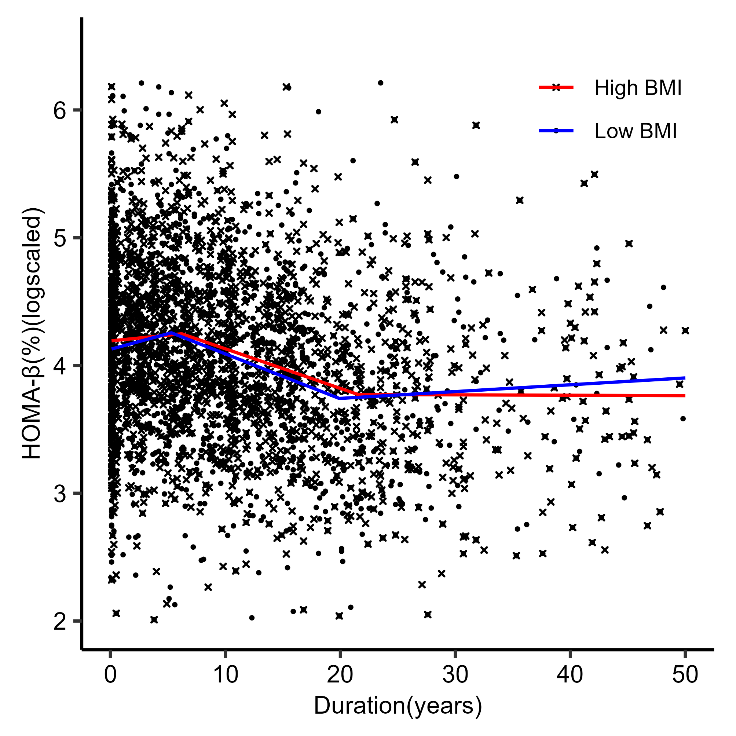


Supplementary Figure 3. HOMA-β Change in patients with high or low BMI

4. We divided 2,898 patients according to Metabolic Syndrome (MetS). We observed patients with MetS had higher HOMA-β levels at initial diagnosis than those without MetS (67.59% vs 52.98%). There were no statistically significant differences in the inflection points or decline rates at each stage between the two groups (Supplementary Table 4, Supplementary Figure 4).

Supplementary Table 4. HOMA-β change patterns in patients with or without MetS

|  | With MetS  (n=1956) | Without MetS  (n=942) | P |
| --- | --- | --- | --- |
| Phase 1 |  |  |  |
| HOMA-β at diagnosis（%） [95% CI] ^§^ | 67.59 [66.44, 68.76] | 52.98 [51.36, 54.67] | 0.000* |
| β1 [95% CI] | 0.0267 [-0.0097, 0.0630] | 0.0714[-0.0184, 0.1613] | 0.283 |
| HOMA-β change per year（%）[95% CI]^†^ | 2.70 [-0.97, 6.51] | 7.42 [-1.82, 1.75] | 0.282 |
| Phase 2 |  |  |  |
| Break point（year）[95% CI] | 4.50 [1.89, 7.11] | 3.10 [0.81, 5.39] | 0.456 |
| β2 [95% CI] | -0.0278 [-0.0374, -0.0183] | -0.0338 [-0.0457, -0.0218] | 0.538 |
| HOMA-β change per year（%）[95% CI] ^†^ | -2.75 [-3.68, -1.81] | -3.33% [-4.47%, -2.16%] | 0.538 |
| Phase 3 |  |  |  |
| Break point（year）[95% CI] | 20.27 [14.14, 26.40] | 21.78 [16.59, 26.97] | 0.683 |
| β3 [95% CI] | -0.0036 [-0.0133, 0.0061] | 0.0119 [-0.0067, 0.0306] | 0.265 |
| HOMA-β change per year（%）[95% CI] ^†^ | -0.36 [-1.32, 0.61] | 1.20 [-0.67, 3.11] | 0.265 |

Note:§exponential of intercept taken to show estimated HOMA-β at diagnosis; †calculated from the exponential of β (the regression slope) -1;***p＜0.001.


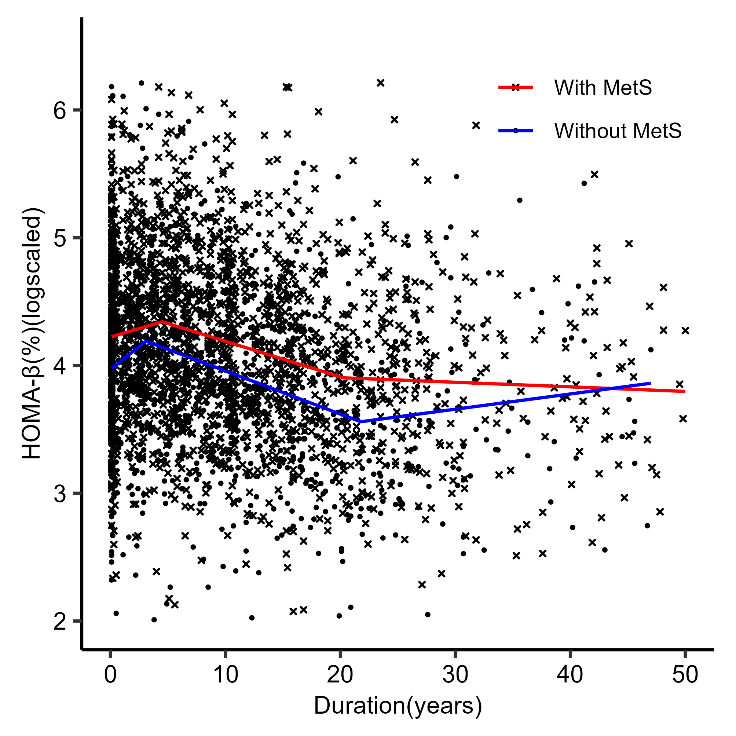


Supplementary Figure 4. HOMA-β Change in patients with or without MetS

5. We divided 2,898 patients according to insulin use. We observed patients who did not use insulin had higher HOMA-β levels than those who did (65.98% vs 51.09%). There were no statistically significant differences in the decline rates at each stage between the two groups (Supplementary Table 5, Supplementary Figure 5).

Supplementary Table 5. HOMA-β change patterns in patients using or not using insulin treatment

|  | Use Insulin  (n=936) | Not Use Insulin  (n=1962) | P |
| --- | --- | --- | --- |
| Phase 1 |  |  |  |
| HOMA-β at diagnosis（%） [95% CI] ^§^ | 51.09 [50.49, 51.69] | 65.98 [65.52, 66.44] | 0.000* |
| β1 [95% CI] | 0.0913 [-0.2130, 0.3956] | 0.0219[-0.0045, 0.0482] | 0.243 |
| HOMA-β change per year（%）[95% CI]^†^ | 9.57 [-19.3, 48.5] | 2.21 [-0.45, 4.94] | 0.243 |
| Phase 2 |  |  |  |
| Break point（year）[95% CI] | 1.50 [-1.81, 4.81] | 5.70 [3.31, 8.09] | 0.023* |
| β2 [95% CI] | -0.0318 [-0.0438, -0.0193] | -0.0305 [-0.0405, -0.0210] | 0.786 |
| HOMA-β change per year（%）[95% CI] ^†^ | -3.19 [-4.39, -1.92] | -3.06% [-4.06, -2.09] | 0.786 |
| Phase 3 |  |  |  |
| Break point（year）[95% CI] | 20.06 [14.46, 25.67] | 20.90 [15.86, 25.94] | 0.678 |
| β3 [95% CI] | 0.0063[-0.0062, 0.0188] | 0.0014 [-0.0115, 0.0144] | 0.647 |
| HOMA-β change per year（%）[95% CI] ^†^ | 0.62 [-0.63, 1.90] | 0.14 [-0.16, 1.43] | 0.647 |

Note:§exponential of intercept taken to show estimated HOMA-β at diagnosis; †calculated from the exponential of β (the regression slope) -1;***p＜0.001.


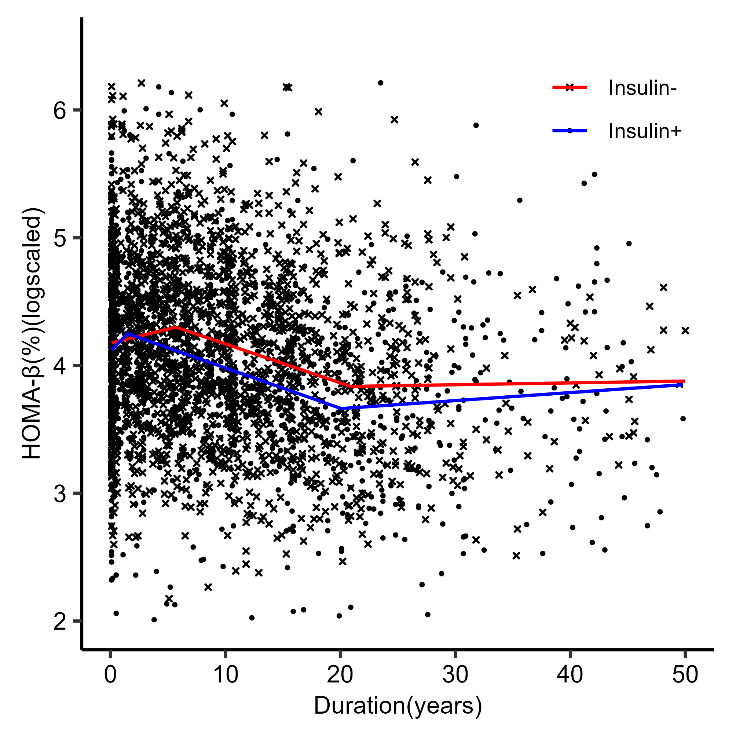


Supplementary Figure 5. HOMA-β Change in patients using or not using insulin treatment
